# Interim Estimates of 2025–26 Seasonal Influenza Vaccine Effectiveness — California, October 2025–January 2026

**DOI:** 10.15585/mmwr.mm7509a3

**Published:** 2026-03-12

**Authors:** Sophie Zhu, Joshua Quint, Tomás M. León, Nancy J. Li, Shantel Muldrew, Charsey Porse, Brendan Flannery, Sascha Ellington, Erin L. Murray, Darpun Sachdev, Kalyani McCullough, Robert Schechter

**Affiliations:** ^1^California Department of Public Health, Richmond, California; ^2^Influenza Division, National Center for Immunization and Respiratory Diseases, CDC.

**Summary**

**What is already known about this topic?**

Influenza vaccine effectiveness (VE) varies annually by season, antigenic similarity between vaccine and circulating viruses, and patient characteristics. VE can be estimated using paired laboratory surveillance and vaccination data. Annual influenza vaccination is recommended; U.S. influenza vaccines remain available for persons aged ≥6 months.

**What is added by this report?**

In California, estimated influenza VE against laboratory-confirmed influenza for all age groups in California during October 2025–January 2026 was 33% (32% against influenza A and 47% against influenza B).

**What are the implications for public health practice?**

State reporting requirements for laboratory surveillance and vaccination data allow for early-season influenza VE estimates. Influenza vaccination is recommended for eligible persons while seasonal influenza viruses are circulating. State-level interim VE estimates provide information to public health officials to facilitate timely local public health actions for prevention and treatment.

## Abstract

Interim estimates of state-level influenza vaccine effectiveness (VE) can help guide timely local public health actions for prevention and treatment of influenza. Linked influenza vaccination and public health influenza surveillance data from California allowed estimation of interim influenza VE by comparing the odds of seasonal influenza vaccination among persons who received positive and negative influenza test results reported to the California Department of Public Health (CDPH) using a case-control study design. During October 1, 2025–January 31, 2026, a total of 952,765 influenza laboratory test results were reported to CDPH. These data were analyzed, including results for 86,369 (9%) persons with receipt of a positive influenza test result (case-patients) and 866,396 (91%) with receipt of a negative test result (control patients). Overall, 22% of case-patients and 27% of control patients were vaccinated against influenza. Interim VE against any influenza was 33% for all age groups, 39% for children and adolescents aged 6 months–17 years, and 22% for adults aged ≥65 years; VE was 32% against a positive influenza A test result, and 47% against a positive influenza B test result. These results suggest that influenza vaccination was associated with reduced odds for laboratory-confirmed influenza among children and adults. CDPH recommends annual influenza vaccination for all persons aged ≥6 months to reduce the risk for influenza and influenza-associated adverse health outcomes.

## Introduction

During October 1, 2025–January 31, 2026, influenza virus infection has caused approximately 22–38 million illnesses, 280,000–590,000 hospitalizations, and 12,000–60,000 deaths across the United States (*1*). In California, influenza activity during the 2025–26 season has been comparable to that in the rest of the United States and has been characterized by moderate disease severity. Influenza vaccines reduce the risk for influenza complications, and antiviral treatment decreases risk for severe disease among persons who are hospitalized, have severe or progressive illness, or are at increased risk for influenza-associated complications.

Seasonal influenza vaccine effectiveness (VE) varies by season, antigenic similarity between vaccine and circulating viruses, and patient characteristics (*2*). Early reports of circulation of antigenically drifted influenza A(H3N2) subclade K viruses raised concerns about potentially low seasonal influenza VE; 92% of genetically characterized viruses in the United States were subclade K *(3)*. However, initial estimates suggested protection against A(H3N2) hospitalization (*4*).

Since the 2023–24 influenza season, the California Department of Public Health (CDPH) has estimated influenza VE in California using linked laboratory reporting and state vaccination data (*5*,*6*). Reporting of positive influenza laboratory test results in California began on October 1, 2019, and negative results have been reported since June 15, 2023. Since January 1, 2023, California state law has required providers to document administered influenza vaccines in California’s immunization information system (IIS), permitting estimation of VE against circulating influenza viruses to guide timely local public health action for prevention and treatment of influenza. This report provides interim estimates of 2025–26 seasonal influenza VE against a positive laboratory test result for influenza A or B in California during October 2025–January 2026.

## Methods

### Data Source and Study Design

VE against laboratory-confirmed influenza was estimated using a case-control design comparing the odds of current season influenza vaccination among persons aged ≥6 months with receipt of a positive influenza test result (case-patients) and those with a negative result (control patients). All persons who received testing for influenza using molecular nucleic acid amplification tests at laboratory, hospital, pharmacy, ambulatory, or community-based testing facilities in California and were reported to CDPH during October 1, 2025–January 31, 2026, were eligible for inclusion. Persons who received a positive SARS-CoV-2 test result were not systematically excluded.

### Data Analysis

Information on patient age, race and ethnicity, county of residence, week of specimen collection, and influenza virus type and subtype results (if available) were extracted from influenza laboratory test reports. Subtyping was performed primarily by local public health laboratories and CDPH, as well as clinical laboratories; approximately 10% of influenza A–positive samples at these laboratories were subtyped. Influenza A samples were selected for sequencing based on sample cycle threshold (an indirect measure of the concentration of virus in the sample) and regional representativeness. As previously reported (*5*,*6*), influenza test results and vaccination records were linked using fuzzy matching.* Among persons with receipt of more than one influenza test result during a season, the earliest positive test result was used to identify influenza case-patients, and among persons who never received a positive test result, the earliest negative test result was used to identify control patients. Negative results were considered likely to be underreported because the level of test positivity (≥50%) was inconsistent with trends for laboratories with comparable test volume and statewide trends; therefore, results from laboratories that reported ≥50% positive influenza test results and those that did not report consistently (i.e., reported varying results from week to week) were excluded. These excluded results represented approximately 5% of total laboratory reports. A person was considered to be vaccinated against influenza if vaccination records from California’s IIS documented receipt of ≥1 dose of 2025–26 seasonal influenza vaccine ≥14 days before influenza testing during August 1, 2025–January 31, 2026. Persons who were vaccinated <14 days before their test date were excluded.

### VE Calculation

Adjusted VE against laboratory-confirmed influenza was estimated as (1 − adjusted odds ratio [aOR ] × 100%), where aOR is the odds of vaccination among influenza test-positive case-patients compared with that among test-negative control patients. Using mixed-effects logistic regression, estimates were adjusted for age and race and ethnicity as fixed effects, and for specimen collection week and county of residence as random effects. Separate analyses were conducted to estimate VE by influenza type (A or B), age group, and vaccine type among certain age groups. All analyses were performed using R software (version 4.5.1; R Foundation). This activity was reviewed by CDPH and CDC, deemed not research, and was conducted consistent with applicable federal law and CDC policy.^†^

## Results

### Influenza Test Results and Virus Type Among Those who Received Positive Results

During October 1, 2025–January 31, 2026, a total of 952,765 influenza laboratory test results meeting inclusion criteria were reported to CDPH, including 86,369 (9%) positive and 866,396 (91%) negative test results. Among positive influenza test results, 82,763 (96%) were influenza type A, and 3,606 (4%) were type B (Table 1). Among 8,071 (10%) influenza A–positive results with subtype information available, 1,954 (24%) were A(H1N1)pdm09, and 6,117 (76%) were A(H3N2). Among 116 A(H3N2) viruses sequenced, 108 (93%) were subclade K, similar to national patterns.

**TABLE 1:** Number and percentage of patients who received positive and negative influenza test results, by demographic characteristics, influenza virus type, and vaccination status — California, October 2025–January 2026

Characteristic	Total	Influenza test result, no. (%)	
		Positive (case-patients)	Negative (control patients)
**Total**	**952,765 (100.0)**	**86,369 (9.0)**	**866,396 (91.0)**
**Median age, yrs (IQR)**	**44 (18–69)**	23 (9–51)	46 (20–70)
**Race**			
American Indian or Alaska Native	**5,442 (0.6)**	457 (0.5)	4,985 (0.6)
Asian	**82,867 (8.8)**	9,489 (11.0)	73,378 (8.5)
Black or African American	**73,554 (7.7)**	5,126 (5.9)	68,428 (7.9)
Native Hawaiian or Pacific Islander	**6,809 (0.7)**	636 (0.7)	6,173 (0.7)
White	**397,449 (41.7)**	30,767 (35.6)	366,682 (42.3)
Other	**223,163 (23.4)**	23,412 (27.1)	199,751 (23.1)
Unknown	**162,481 (17.1)**	16,482 (19.2)	146,999 (16.9)
**Ethnicity**			
Hispanic or Latino	**252,860 (26.5)**	26,505 (30.7)	226,355 (26.1)
Not Hispanic or Latino	**529,824 (55.6)**	43,621 (50.5)	486,203 (56.1)
Unknown	**170,081 (17.9)**	16,243 (18.8)	153,838 (17.8)
**Sex (n = 952,665)**			
Female	**519,184 (54.5)**	46,565 (53.9)	472,619 (54.6)
Male	**432,544 (45.4)**	39,647 (45.9)	392,897 (45.3)
Unknown	**937 (0.1)**	154 (0.2)	783 (0.1)
**Influenza virus type**			
A	—	82,763 (95.8)*	—
B	—	3,606 (4.2)	—
**Month vaccinated**			
Oct 1–31	**26,862 (14.3)**	207 (10.7)	26,655 (14.3)
Nov 1–30	**48,772 (25.3)**	662 (16.7)	48,110 (25.3)
Dec 1–30	**81,141 (30.3)**	6,258 (20.6)	74,883 (31.6)
Jan 1–31	**97,380 (32.2)**	11,561 (23.0)	85,819 (34.0)
Total	**254,155 (26.7)**	18,688 (21.6)	235,467 (27.2)
**No. of days between vaccination and receipt of test result, median (IQR)**	**68 (42–96)**	87 (61–108)	67 (41–95)
**Receipt of high-dose, adjuvanted, or recombinant vaccine (patients aged ≥65 yrs)**			
Received	**112,699 (92.8)**	5,173 (91.7)	107,526 (92.9)
Did not receive	**8,530 (7.2)**	465 (8.3)	8,065 (7.1)

* Among 8,071 (10%) influenza A–positive results with subtype information available, 1,954 (24%) were A(H1N1)pdm09, and 6,117 (76%) were A(H3N2). Among 116 A(H3N2) viruses sequenced, 108 (93%) were subclade K.

### Percentage of Positive and Negative Influenza Test Results Among Vaccinated Patients

Overall, 254,155 (27%) persons had documentation of receipt of the 2025–26 influenza vaccine, including 18,688 (22%) persons with receipt of a positive influenza test result and 235,467 (27%) with receipt of a negative result. A majority of vaccinated adults aged ≥65 years (112,699 of 121,229; 93%) had documentation of receiving a preferentially recommended vaccine (high-dose, adjuvanted, or recombinant vaccine), per the recommendation from CDC’s Advisory Committee on Immunization Practices (*7*).

### VE

Adjusted VE was 33% against receiving a positive influenza (A or B) test result, 32% against receiving a positive influenza A test result, and 47% against receiving a positive influenza B test result (Table 2). By age group, VE was 39% among persons aged <18 years, 34% among adults aged 18–49 years, 31% among adults aged 50–64 years, and 22% among adults aged ≥65 years. Among children and adolescents aged 2–17 years who were eligible for live attenuated influenza vaccine (LAIV), VE was 55% for LAIV and 39% for standard-dose inactivated influenza vaccine. VE by vaccine product type among adults aged ≥65 years was 39% for recombinant vaccine, 22% for adjuvanted and high-dose vaccines, and 16% for standard-dose inactivated influenza vaccines.

**TABLE 2:** Vaccine effectiveness among patients who received a 2025–26 influenza vaccine, by influenza test result, influenza virus type and vaccine received, and age group — California, October 2025–January 2026

Influenza virus type, vaccine received, and age group	Influenza test result				Vaccine effectiveness*
	Positive (case-patients)		Negative (control patients)		
	Total	Vaccinated, no. (row %)^†^	Total	Vaccinated, no. (row %)^†^	% (95% CI)
**Any influenza (A or B)**	**86,369**	18,688 (22)	**866,396**	235,467 (27)	33 (32–34)
6 mos–17 yrs	**36,726**	6,897 (19)	**198,561**	40,248 (20)	39 (37–41)
2–17 yrs^§^	**32,614**	5,897 (18)	**153,836**	29,697 (19)	40 (38–42)
LAIV	**—**	170 (3)^¶^	**—**	1,166 (4)^¶^	55 (46–62)
IIV–SD	**—**	5,472 (93)^¶^	**—**	27,003 (96)^¶^	39 (36–41)
18–49 yrs	**27,660**	4,074 (15)	**263,387**	45,001 (17)	34 (31–36)
50–64 yrs	**8,513**	2,079 (24)	**131,337**	34,627 (26)	31 (27–34)
≥65 yrs**	**13,470**	5,638 (42)	**273,111**	115,591 (42)	22 (19–25)
IIV–HD	**—**	3,673 (65)^¶^	**—**	76,986 (67)^¶^	22 (19–26)
RIV	**—**	116 (2)^¶^	**—**	2,734 (2)^¶^	39 (26–49)
aIIV	**—**	1,384 (25)^¶^	**—**	27,806 (24)^¶^	22 (17–27)
IIV–SD	**—**	465 (8)^¶^	**—**	8,065 (7)^¶^	16 (7–23)
**Influenza A**	**82,763**	18,145 (22)	**866,396**	235,467 (27)	32 (31–33)
6 mos–17 yrs	**34,806**	6,591 (19)	**198,561**	40,248 (20)	39 (37–40)
18–49 yrs	**26,278**	3,900 (15)	**263,387**	45,001 (17)	33 (30–35)
50–64 yrs	**8,326**	2,045 (25)	**131,337**	34,627 (26)	31 (27–34)
≥65 yrs	**13,353**	5,609 (42)	**273,111**	115,591 (42)	22 (19–25)
**Influenza B**	**3,606**	543 (15)	**866,396**	235,467 (27)	47 (42–52)
6 mos–17 yrs	**1,920**	306 (16)	**198,561**	40,248 (20)	46 (39–53)
18–49 yrs	**1,382**	174 (13)	**263,387**	45,001 (17)	47 (38–55)
50–64 yrs	**187**	34 (18)	**131,337**	34,627 (26)	47 (23–63)
≥65 yrs	**117**	29 (25)	**273,111**	115,591 (42)	56 (32–71)

**Abbreviations:** aIIV = adjuvanted inactivated influenza vaccine; IIV-HD = inactivated influenza vaccine, high dose; IIV-SD = inactivated influenza vaccine, standard dose; LAIV = live attenuated influenza vaccine; RIV = recombinant influenza vaccine.

* Adjusted for age (natural cubic spline) and race and ethnicity as fixed effects and specimen collection week, and county of residence as random effects using mixed-effects logistic regression.

^†^ Row percent, except as indicated (percentage who received each vaccine calculated from among persons vaccinated).

^§^ Children aged <2 years are not eligible for LAIV and were not included in this analysis. All vaccines, including IIV-SD, were compared with no influenza vaccination as the referent group.

^¶^ Calculated as percentage of persons vaccinated (column %).

** Adults aged ≥65 years are preferentially recommended to receive IIV-HD, RIV, or aIIV (https://dx.doi.org/10.15585/mmwr.mm7432a2). All vaccines, including IIV-SD, were compared with no influenza vaccination as the referent group.

## Discussion

Analysis of California surveillance data from influenza vaccination and laboratory reporting systems suggests that seasonal influenza vaccination provided protection against laboratory-confirmed influenza across all age groups during October 2025–January 2026. VE was higher among younger age groups and declined with increasing age, was higher against influenza B viruses, and was slightly higher for LAIV among children aged 2–17 years. VE estimates are consistent with California influenza VE from previous years (*5*,*6*). Influenza vaccination has been demonstrated to reduce the risk for influenza illness and severe outcomes associated with influenza, including hospitalization and death among children and adults (*8*,*9*). Influenza vaccination was recommended for the 2025–26 influenza season for all persons aged ≥6 months (*7*).

Interim VE estimates against influenza A–positive test results suggest that influenza vaccination has provided protection against antigenically drifted A(H3N2) clade K viruses that have predominated in California and in other areas of the United States during the current season. Estimates were consistent with interim VE estimates from Canada (40% overall against A[H3N2]) (*10*).

California’s influenza vaccination and laboratory reporting requirements permit interim VE estimates using routine surveillance data; these estimates contribute state-level data to national estimates and can be considered alongside CDC VE surveillance systems (*6*). Interim VE estimates were shared with California local health jurisdictions during January 2026 to help guide influenza vaccination and treatment messaging and actions.

### Limitations

The findings in this report are subject to at least five limitations. First, VE estimates are preliminary and are limited to California; multiple influenza viruses circulate nationally, and VE might vary geographically. Second, influenza test result and vaccination data might be incomplete and affect the calculated VE. Third, children and adolescents aged 6 months–8 years who received only 1 dose of 2025–26 vaccine during their first influenza season, when 2 doses are required to be considered fully vaccinated (*7*), might be misclassified as fully vaccinated, which could have resulted in an underestimate of VE. Fourth, data on clinical outcomes (e.g., hospitalization or death) and testing settings (e.g., outpatient, inpatient, or intensive care unit) were not available to compare VE by setting. Finally, other potential sources of confounding, including previous influenza vaccination, preexisting conditions, and health-seeking behavior, were not controlled for in the analyses.

### Implications for Public Health Practice

VE estimates from surveillance systems based on different approaches contribute to evaluation of influenza vaccine benefit. Interim estimates of state-level influenza VE calculated while influenza viruses are still circulating can provide information to public health officials to facilitate timely local public health actions for prevention and treatment of influenza, such as reinforced vaccination messaging, and contribute data to national estimates.

Annual influenza vaccination is recommended by the Advisory Committee on Immunization Practices and CDPH; influenza vaccines remain available for persons aged ≥6 months. Eligible persons who have not yet been vaccinated are recommended to receive influenza vaccine while influenza viruses are circulating. Influenza vaccines protect against influenza and its complications, and early treatment with influenza antiviral medications decreases risk for severe disease and hospitalization.

## Footnotes

* California influenza testing and immunization registries were matched using a probabilistic algorithm with exact match for date of birth and fuzzy match (similar and partially matching, but not identical) with a 95% cutoff for first name, last name, and county of residence.

† 45 C.F.R. part 46, 21 C.F.R. part 56; 42 U.S.C. Sect. 241(d); 5 U.S.C. Sect. 552a; 44 U.S.C. Sect. 3501 et seq.
